# Sociodemographic factors associated with knowledge and attitudes of Peruvian dental interns about dental shade selection

**DOI:** 10.1186/s12909-023-04971-1

**Published:** 2023-12-19

**Authors:** Delia Vásquez-Pérez, Gissela Briceño-Vergel, Marysela Ladera-Castañeda, Nancy Córdova-Limaylla, Luis Cervantes-Ganoza, Elizabeth Paucar-Rodríguez, Clifford Allen-Revoredo, Miriam Castro-Rojas, César Cayo-Rojas

**Affiliations:** 1 Universidad Privada San Juan Bautista, School of Stomatology, Lima, Peru; 2 Universidad Nacional Federico Villarreal, Postgraduate School, Research Team “Salud Pública – Salud Integral”, Lima, Peru; 3 Faculty of Stomatology, Universidad Inca Garcilaso de la Vega, Lima, Peru

**Keywords:** Attitude, Associated factors, Color, Color matching, Dental education, Dental interns, Knowledge, Shade selection

## Abstract

**Background:**

The dentist should have a thorough knowledge of the science, protocols and procedures for dental shade selection in order to mimic dental tissue with restorative materials, respecting the individual needs and attitudes of each patient and providing them with a pleasant and esthetically acceptable appearance. The present study evaluated the knowledge and attitudes of dental interns from two Peruvian universities about dental shade selection and its relationship with sociodemographic factors.

**Methods:**

The present cross-sectional and analytical study was carried out on Peruvian dental interns from the Universidad Nacional Federico Villarreal and the Universidad Privada de San Juan Bautista during May to June 2022. Two validated questionnaires on knowledge and attitudes about dental shade selection were used. A Poisson regression model with robust variance using the adjusted prevalence ratio (APR) was used to assess the influence of the variables: age group, sex, place of origin and type of university, considering a significance level of *p*<0.05.

**Results:**

The results showed that 40.8% (95% CI: 34.0% - 47.6%) of the interns presented unfavorable attitudes while 90.1% (95% CI: 86.0% - 94.2%) presented insufficient knowledge. It was also found that dental interns under 29 years of age were 32% less likely to have unfavorable attitudes (APR = 0.68, 95% CI 0.48 - 0.96) and 11% more likely to have insufficient knowledge (APR = 1.11, 95% CI 1.01 - 1.24) about tooth shade selection compared to dental interns aged 29 years and older. Finally, women were 55% more likely to have unfavorable attitudes about tooth shade selection compared to men (APR = 1.55; 95% CI: 1.08 - 2.22).

**Conclusion:**

The majority of dental interns presented insufficient knowledge and less than half had unfavorable attitudes about dental shade selection. In addition, being a student under 29 years of age was a risk factor for presenting insufficient knowledge and at the same time constituted a protective factor for presenting unfavorable attitudes. Likewise, being a female student was a risk factor for presenting unfavorable attitudes about dental shade selection.

## Background

The importance of esthetics nowadays and people's concern for physical beauty together with a harmonious smile goes beyond being a luxury and has become a necessity for social and cultural acceptance, and can interfere psychologically affecting personal self-esteem [1–4].

The success of an esthetic dental treatment is determined primarily on the basis of four determining factors: position, contour, texture and shade [2, 5]. Shade selection becomes important in esthetic dentistry because of the complexity of harmonizing artificial restorations with adjacent teeth. Although shade may not be critical to physiologic function, it plays a major role in patient acceptance [2, 3].

The two main methods for shade selection are the conventional visual method that uses tabs of generally tooth-shaped shades (shade guide) and the instrumental method that uses shade measurement devices such as the spectrophotometer [6].

Although shade is a relevant esthetic parameter in the final result of the restorations, very often mistakes are made in its choice because it is a subjective assessment in which various factors may interfere at the time of shade selection [7] such as the type and intensity of the light source, the season, the angle of incidence, the patient's clothing, age, sex, time of day, among others [2].

The dentist should have a thorough knowledge of the science, protocols and procedures for dental shade selection in order to mimic dental tissue with restorative materials, respecting the individual needs and attitudes of each patient and providing them with a pleasant and esthetically acceptable appearance [6, 8, 9]. For this reason, it is expected that dental interns, having received this training in clinical courses within the university, will handle these protocols when performing their pre-professional internships in hospitals. It should be noted that the dental intern is a student in the final phase of his or her training, enrolled in a Peruvian university, who carries out pre-professional internships under the strict supervision of the university, whose activities are formalized through a resolution of officialization from the General Office of Human Resources Management of the Ministry of Health, which does not generate an employment relationship with the entity where the internship is carried out [10].

Therefore, the present study aimed to evaluate the knowledge and attitudes of dental interns from two Peruvian universities about dental shade selection and its relationship with sociodemographic factors.

## Methods

### Study design

The present study was written according to the STrengthening the Reporting of OBservational studies in Epidemiology (STROBE) guidelines [11]. In addition, this observational, prospective, cross-sectional and analytical study was conducted in Peruvian dental interns of the Universidad Nacional Federico Villarreal (UNFV) and the Universidad Privada de San Juan Bautista (UPSJB) during the months of May to June 2022.

### Population and selection of participants

The total population consisted of 248 dental interns (128 from the UPSJB and 120 from the UNFV). It was not necessary to calculate a sample size since the entire population was surveyed according to the selection criteria. The target population was N = 201 participants (127 from UPSJB and 74 from UNFV).

#### Inclusion criteria

- Dental interns from UPSJB and UNFV.
- Dental interns enrolled in the period 2022-1.
- Dental interns who gave voluntary informed consent.

#### Exclusion criteria

- Dental interns who did not complete the questionnaire.

### Variables

The dependent variable was knowledge and attitudes about dental shade selection and the independent variable was age [12] and sex [13–17], while the covariates were place of origin [18] and type of university [18].

### Instrument design, validation and application

To assess the knowledge about dental shade selection, a 12-question questionnaire was developed based on the research of Sambandam et al [5] and complemented with literature available in databases such as Scopus and Pubmed. Three experts in biomaterials research validated the pertinence, objectivity, relevance, timeliness and clarity of the content of the questionnaire, obtaining an acceptable Aiken's V (V = 0.86, CI: 0.81 - 0.89). According to the principal component factor analysis, a single dimension was obtained with acceptable values for Bartlett's test of sphericity (*p*<0.001) and the Kaiser-Mayer-Olkin measure (KMO = 0.828). Cronbach's Alpha coefficient was used to evaluate the internal consistency of the questionnaire, obtaining a value of 0.76 (95% CI: 0.70 - 0.80) which was considered acceptable. In addition, to evaluate the reproducibility of the questionnaire, the questions were asked to 30 dental interns at two different times in a period of 10 days and altering the order of the questions to avoid memory bias [18, 19]. The intraclass correlation coefficient (ICC) of the scores obtained (ICC = 0.90; 95% CI: 0.78 - 0.95) was acceptable. The score given to each correct answer was 1 point and to each incorrect answer 0 points. The total score was categorized from 0 to 6 points as poor level, from 7 to 8 points as fair level and from 9 to 12 points as good level. Knowledge was then dichotomised as insufficient if it was poor and fair (0 to 8 points) and sufficient if it was good (9 to 12 points). The cut-off point was validated using Livingston's K^2^ coefficient, which was acceptable at 0.853.

In order to evaluate attitudes about dental shade selection, a questionnaire was modified using literature available in databases such as Scopus and Pubmed [7]. This consisted of 9 questions and the answers were on a Likert scale (never = 1, sometimes = 2, often = 3 and always = 4). The scale content was validated, obtaining an acceptable Aiken's V (V = 0.86, CI: 0.82 - 0.90). According to the principal component factor analysis, a single dimension was obtained with acceptable values for Bartlett's test of sphericity (*p*<0.001) and the Kaiser-Mayer-Olkin measure (KMO = 0.778). Cronbach's Alpha coefficient was used to evaluate the internal consistency of the questionnaire, obtaining a value of 0.74 (IC 95%: 0.68 – 0.79) which was considered acceptable. In addition, to evaluate the reproducibility of the questionnaire, the questions were asked to 30 dental interns at two different times in a period of 10 days and altering the order of the questions to avoid memory bias. The reproducibility of the questionnaire was very good, with an ICC = 0.98 (95% CI: 0.97 - 0.99). The total score was categorized from 9 to 18 points as poor level, from 19 to 27 points as fair level and from 28 to 36 points as good level. Attitude was then dichotomised as unfavorable attitude if they were poor and fair (9 to 27 points) and favorable attitude if they were good (28 to 36 points). The cut-off point was validated by Livingston's K^2^ coefficient, which was acceptable at 0.755.

### Procedure

The questionnaires were prepared using Google Forms and distributed in a self-administered manner through institutional e-mails and WhatsApp Messenger®. The first page contained the informed consent form explaining the objective of the study and the risk/benefit. It was also emphasized that respondents could withdraw from the study at any time if they felt any discomfort when answering the questions. The institutional e-mail addresses of the principal researcher and the ethics committee were also provided in case there were any doubts. The principal researcher (DVP) collected the information and tabulated the data in a Microsoft Excel 2019 spreadsheet. All researchers had access to this information, which was stored on a digital handheld device with a password to maintain confidentiality. All printed data was destroyed for security. At the end of the study, the results were sent to those who requested the information to the principal researcher via email. The survey was available online from May 1 to June 30, 2022.

### Statistical analysis

Statistical Package for the Social Sciences (SPSS) version 28.0 was used for data analysis. For the descriptive analysis, the results were presented with absolute and relative frequencies for qualitative variables and the mean and standard deviation for quantitative variables. For bivariate analysis, Pearson's chi-square test was applied and for expected values less than 5, Fisher's exact test was applied. For multivariate analysis, a Poisson regression model with robust variance using adjusted prevalence ratio (APR) was used considering possible confounding variables. The significance level of all statistical analyses was *p*<0.05.

### Bioethical considerations

The Institutional Research Ethics Committee of the Universidad Privada San Juan Bautista approved the project with resolution No. 567-2022-CIEI-UPSJB, since the bioethical principles of freedom, confidentiality, non-maleficence and respect for medical research on human beings set forth in the Declaration of Helsinki were respected [20].

## Results

**Fig. 1:**
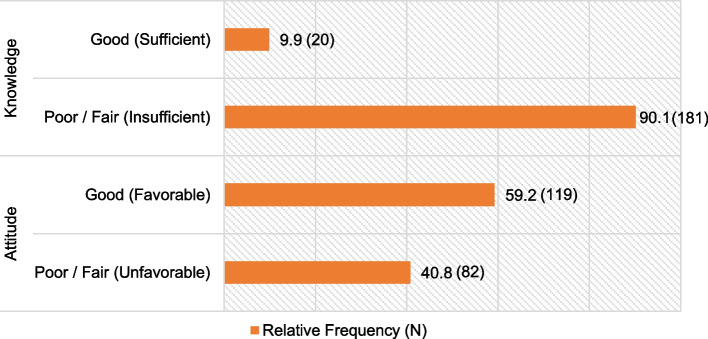
Relative frequencies of attitudes and knowledge about dental shade selection in dental interns

The response rate of the total number of participants surveyed was 81.04%. The mean age was 30.5 ± 9.3 years. 52.2% were under 29 years of age and 55.7% were female. 64.7% were from the capital city and 63.2% of the total studied at a private university [Table 1]. In addition, of the total number of participants surveyed, 40.8% (CI: 34.0% - 47.6%) had unfavorable attitudes (poor and fair), this being the category of interest; while 90.1% (CI: 86.0% - 94.2%) had insufficient knowledge (poor and fair), this being the category of interest [Fig. 1].

**Table 1:** Characterization of sociodemographic factors of dental interns

**Variable**	**Category**	**Frequency**	**Percentage**
**Age group**	< 29 years	105	52.2
	≥ 29 years	96	47.8
**Sex**	Female	112	55.7
	Male	89	44.3
**Place of origin**	Capital	130	64.7
	Province	71	35.3
**Type of university**	Private	127	63.2
	Public	74	36.8
**Age**	**Mean**	**Median**	**SD**
	30.5	28.0	9.3

*SD* Standard Deviation

It was observed that attitudes about tooth shade selection were significantly associated with the variable age group and sex (*p* = 0.024 and *p* = 0.016; respectively). While, the level of knowledge about tooth shade selection was significantly associated with age group (*p* = 0.036) [Table 2].

**Table 2:** Sociodemographic variables associated with attitudes and knowledge of dental interns about dental shade selection.

**Variable**	**Category**	**Attitudes**		p*	**Knowledge**		p*
		**Unfavorable**	**Favorable**		**Insufficient**	**Sufficient**	
		**f (%)**	**f (%)**		**f (%)**	**f (%)**	
**Age group**	< 29 years	35 (33.3)	70 (66.7)	0.024*	99 (94.3)	6 (5.7)	0.036*
	≥ 29 years	47 (49.0)	49 (51.0)		82 (85.4)	14 (14.6)	
**Sex**	Female	54 (48.2)	58 (51.8)	0.016*	101 (90.2)	11 (9.8)	0.945
	Male	28 (31.5)	61 (68.5)		80 (89.9)	9 (10.1)	
**Place of origin**	Capital	57 (43.8)	73 (56.2)	0.234	118 (90.8)	12 (9.2)	0.645
	Province	25 (35.2)	46 (64.8)		63 (88.7)	8 (11.3)	
**Type of university**	Private	54 (42.5)	73 (57.5)	0.515	114 (89.8)	13 (10.2)	0.859
	Public	28 (37.8)	46 (62.2)		67 (90.5)	7 (9.5)	

*f* Absolute frequency; * based on Pearson's Chi-square, significant association (*p*<0.05)

Regarding the attitudes towards tooth shade selection, according to the frequency and the measure of central tendency of the responses, it was observed that they always considered using natural light for the shade selection of a fixed prosthesis and that the dental office should have soft tones, and they also always considered it useful to take a 5 to 10 second break during tooth shade selection (A1, A4 and A8). They also always considered doing prophylaxis, covering whiskers and removing lipsticks before tooth shade selection (A2 and A3). On the other hand, the minority always considered the help of an assistant practical and that the patient's clothing should have a soft shade and their teeth should be kept moist during tooth shade selection (A5, A6 and A7). Finally, the majority of students considered that it is never or sometimes useful to use the spectrophotometer for tooth shade selection (A9) [Table 3].

**Table 3:** Descriptive analyses of attitudes about dental shade selection

**Questions**	**Total**			**Never**	**Sometimes**	**Often**	**Always**
	**Mean**	**Median**	**SD**	**f (%)**	**f (%)**	**f (%)**	**f (%)**
**A1.** I consider using natural light for dental shade selection in fixed prostheses.	3.49	4.00	0.76	5 (2.5)	18 (9.0)	51 (25.4)	127 (63.2)
**A2.** I consider it necessary to perform a dental prophylaxis prior to shade selection.	3.62	4.00	0.66	3 (1.5)	11 (5.5)	45 (22.4)	142 (70.6)
**A3.** I find it useful to remove strong colored lipsticks and cover whiskers for dental shade selection.	3.39	4.00	0.92	15 (7.5)	15 (7.5)	48 (23.9)	123 (61.2)
**A4.** I believe that the dental office should have an environment with soft colors so as not to alter the dental shade selection.	3.29	4.00	0.97	16 (8.0)	25 (12.4)	44 (21.9)	116 (57.7)
**A5.** I consider that the patient's clothing should have soft tones so as not to alter the dental shade selection.	2.67	3.00	1.16	42 (20.9)	52 (25.9)	38 (18.9)	69 (34.3)
**A6.** I consider the help of an assistant to be useful for dental shade selection.	2.81	3.00	1.01	19 (9.5)	67 (33.3)	48 (23.9)	67 (33.3)
**A7.** I find it useful to keep the teeth moist during shade selection.	3.09	3.00	1.03	22 (10.9)	31 (15.4)	54 (26.9)	94 (46.8)
**A8.** I consider it useful to take a visual break of 5 to 10 seconds during dental shade selection.	3.32	4.00	0.90	8 (4.0)	35 (17.4)	42 (20.9)	116 (57.7)
**A9**. I consider the use of the spectrophotometer to be useful for dental shade selection.	2.47	2.00	1.12	51 (25.4)	53 (26.4)	48 (23.9)	49 (24.4)

*SD* Standar Deviation, *f* Absolute frequency; *(%)* Percentage

Most of the students were unaware of the importance of handling the theoretical concepts of tooth shade selection (K1). In addition, they mistakenly thought that translucency was the most important for tooth shade selection and did not know the right temperature for shade selection (K2 and K4). Most of them did not know at which time of the appointment the tooth shade should be taken and also believed that the visual method was the ideal method for tooth shade selection (K6 and K9). Finally, they mistakenly believed that isolating the operative field with cotton rolls improved shade selection in anterior teeth (K11) [Table 4].

**Table 4:** Descriptive analyses of knowledge about dental shade selection

**Questions**	**Incorrect**	**Correct**
	**f (%)**	**f (%)**
**K1**. For shade selection, is it necessary to have more skill than knowledge?	121 (60.2)	80 (39.8)
**K2**. Does translucency play the most important role in shade selection?	169 (84.1)	32 (15.9)
**K3.** Does the light source affect shade perception?	24 (11.9)	177 (88.1)
**K4.** Is 5500 K (degrees kelvin) the proper temperature for selecting the dental shade?	117 (58.2)	84 (41.8)
**K5.** Is 5 to 10 seconds the ideal time for dental shade selection?	75 (37.3)	126 (62.7)
**K6.** Is it preferable to make dental shade selection at the end of the dental appointment?	111 (55.2)	90 (44.8)
**K7.** Does the patient's age and sex play a role in dental shade selection?	40 (19.9)	161 (80.1)
**K8.** Is it inappropriate to take patients' opinions into account in dental shade selection?	63 (31.3)	138 (68.7)
**K9.** Is the visual method ideal for dental shade selection?	184 (91.5)	17 (8.5)
**K10**. For the visual method is it preferable to perform dental shade selection with artificial light?	66 (32.8)	135 (67.2)
**K11.** Does isolation of the operating field with cotton rolls improve shade selection in anterior teeth?	127 (63.2)	74 (36.8)
**K12**. For dental shade selection, is the tooth considered by thirds: cervical, middle and incisal?	32 (15.9)	169 (84.1)

*f* Absolute frequency, *(%)* Percentage

When attitudes (unfavorable = 1 and favorable = 0) and knowledge (insufficient = 1 and sufficient = 0) were analysed under an adjusted Poisson regression model with robust variance, it was found that dental interns under 29 years of age were 32% less likely to have unfavorable attitudes about tooth shade selection compared to those over 29 years of age (APR = 0.68, 95% CI: 0.48 - 0.96). In addition, women were found to be 55% more likely to have unfavorable attitudes about tooth color selection compared to men (APR = 1.55; 95% CI: 1.08 - 2.22). Finally, dental interns younger than 29 years were 11% more likely to have insufficient knowledge about tooth shade selection than dental interns aged 29 years and older (APR = 1.11; 95% CI: 1.01 - 1.24) [Table 5].

**Table 5:** Logistic regression model for attitudes and knowledge about dental shade selection according to associated factors

**Factors**		**Crude model**								**Adjusted model**							
		**Unfavorable attitudes**				**Insufficient knowledge**				**Unfavorable attitudes**				**Insufficient knowledge**			
		**PR**	**95% CI**		***p***	**PR**	**95% CI**		***p***	**APR**	**95% CI**		*p	**APR**	**95% CI**		*p
			**LL**	**UL**			**LL**	**UL**			**LL**	**UL**			**LL**	**UL**	
**Age group**	< 29 years	0.68	0.49	0.96	0.026	1.10	1.00	1.21	0.042	0.68	0.48	0.96	0.027	1.11	1.01	1.24	0.048
	≥ 29 years	*Ref.*				*Ref.*				*Ref.*				*Ref.*			
**Sex**	Female	1.53	1.07	2.20	0.021	1.00	0.91	1.10	0.946	1.55	1.08	2.22	0.017	0.99	0.91	1.09	0.974
	Male	*Ref.*				*Ref.*				*Ref.*				*Ref.*			
**Place of origin**	Capital	1.25	0.86	1.80	0.246	1.02	0.93	1.13	0.654	1.17	0.81	1.70	0.403	1.04	0.94	1.15	0.448
	Province	*Ref.*				*Ref.*				*Ref.*				*Ref.*			
**Type of university**	Private	1.12	0.79	1.60	0.520	0.99	0.90	1.09	0.858	0.95	0.67	1.36	0.795	1.00	0.91	1.11	0.930
	Public	*Ref.*				*Ref.*				*Ref.*				*Ref.*			

^*^Adjusted multiple regression model (*p<0.05, significant association)

*APR* Adjusted Prevalence Ratio under Poisson regression model with robust variance, *95% CI* 95% Confidence Interval, *LI* Lower Limit, *UL* Upper Limit

## Discussion

Nowadays, dentistry is faced with patients' demands to meet high esthetic standards [21, 22]. This makes dental shade selection a fundamental step in achieving optimal results. According to Ellakany et al [21], theoretical teaching given to students is not sufficient to achieve better perception in tooth shade selection. Therefore, it is necessary for students to improve their knowledge and skills with a variety of clinical cases during their professional training, especially during the hospital internship, given that the dental intern is a student in the final year of their professional career, who carries out pre-professional healthcare practices with the aim of integrating and reinforcing the competencies previously acquired throughout their training. This dental internship is carried out according to the current curriculum of each university with rotations in first, second and third level health establishments, under the supervision of a professor in dentistry [23, 24]. However, this proved to be a challenge during the COVID-19 pandemic due to the suspension of pre-professional clinical procedures because of the risk of contagion. The present study aimed to evaluate the knowledge and attitudes of dental interns from two Peruvian universities about dental shade selection and its relationship with sociodemographic factors.

The present study showed that 90.1% presented insufficient knowledge (poor and fair) about dental shade selection. This result differs from that reported by Oremosu et al [15] who found that 86.4% of their respondents presented insufficient knowledge (poor and fair). This could be due to the fact that the present study was conducted during the pandemic period. The classes were conducted in a virtual format without the students performing clinical procedures, which may have reduced their motivation in the teaching-learning process [25]. The photographs and audiovisual resources used in virtual education are learning complements that do not replace the actual experience of selecting the dental shade in a patient. This process involves the analysis of shade, environmental conditions, surrounding tissues, optical properties of the teeth and patient compliance, among others [26–29]. Likewise, Enone et al [30] found that 67.5% of the participants in their study had a good knowledge of dental shade selection. This could be due to the fact that the study was applied to dentists with more than 10 years of experience. The present study surveyed dental interns with minor clinical experience. This may have limited their ability to retain knowledge about dental shade selection as it has been reported to increase over time through training, activity, and clinical experience [2, 17]. In the present study, most of the students mistakenly thought that translucency was most important for tooth shade selection and also did not know the appropriate temperature for shade selection. In addition, they did not know at which time of the appointment the tooth shade should be selected and they also wrongly believed that the visual method was the ideal one for this purpose. Finally, they mistakenly believed that isolation of the operative field with cotton rolls improved shade selection in the anterior teeth. This situation could be attributed to the fact that the students did not go into this topic in depth, as they had no training or clinical practice in situ, due to the social isolation during the pandemic [31]. Translucency is not the most important property for tooth shade selection, as it must be considered along with shape, topography, surface texture, lightness, chroma and hue [32]. It is also important to note that the ideal light for tooth shade selection in the clinic is that which is closest to the spectrum of daylight, recommending corrected light sources offering color temperatures of 5000ºK to 6500ºK [33]. It is preferable that the combination and selection of tooth colors is done in the morning, when eye fatigue is minimal [8]. On the other hand, isolation with cotton rolls is intended to keep saliva away from the tooth structure to be restored and to reduce to some extent the aerosols produced during the dental procedure. However, it is not essential for the determination of tooth color, as such a procedure may dehydrate the teeth and result in a whiter appearance [8, 34, 35]. Finally, it is important to note that the selection of tooth shade by visual means may be too subjective, as color perception may be biased due to ambient light, observer experience, fatigue and age, so spectrophotometer measurement has been recommended because it is more accurate and reproducible [33].

Regarding attitudes, the findings of the present study indicated that 40.8% of the inmates presented unfavorable attitudes. In that sense, considering that one of the characteristics that influence the development of attitudes is the cognitive aspect on a given subject [36, 37], probably insufficient knowledge could have influenced the unfavorable attitude found. Oremosu et al [15], Saqib et al [13] and Rozar et al [3] reported that knowledge, depending on the level, can positively or negatively influence students' skills and attitudes in dental shade selection [13, 15, 21]. In addition, most of the students were in favour of always using natural light for tooth shade selection and that the dental office should have soft color shades. Also, most of them always found it useful to prophylaxis the patients, to cover their moustaches, to remove lipsticks before tooth shade selection and to pause for 5 to 10 seconds during this procedure. However, the majority showed a tendency to not always require the help of an assistant, which could be due to the fact that the students, given their lack of clinical experience, are not yet aware of the phenomenon of "eye fatigue" due to the constant stimulation of the nerves involved in color vision, and that the support of a dental assistant is necessary to corroborate the dental color already taken by the dentist [13]. Furthermore, it is particularly striking that the attitude of the students is not good towards the use of the spectrophotometer. This could be due to the fact that the use of this equipment is not strongly encouraged in Peru, due to its high cost and/or the lack of implementation of this equipment in the vast majority of Peruvian universities, thus wasting an objective and useful tool for tooth shade selection [13, 15].

This study found that interns younger than 29 years of age were 11% more likely to have insufficient knowledge about tooth shade selection compared to those over 29 years of age. This may have been due to the fact that the majority of older students may have attended a greater number of lectures and non-instructional courses on the topic of shade selection. Many students are concerned about acquiring up-to-date knowledge that will enable them to develop the skills necessary to meet the patient's needs for esthetic treatments [38, 39]. Also, dental interns younger than 29 years were 32% less likely to have unfavorable attitudes than those aged 29 years or older. This could be because the younger students may have had less family and/or work load [12], which may have created a better predisposition to learn more about novel and interactive clinical procedures. Younger interns probably have a greater concern for more esthetic results as they are generally more concerned with attractive physical appearance than older individuals [40]. Consequently, the findings show that being a student under 29 years of age was a risk factor for presenting insufficient knowledge about dental shade selection and at the same time it constituted a protective factor for presenting unfavorable attitudes about the same topic. This apparent contradiction can be explained by the fact that students under 29 years of age had a good attitude towards learning about esthetic topics inherent to the dental profession, but the desire to learn often did not translate into sufficient knowledge due to multiple factors such as poor study habits, having been taught by non-specialist professors and/or with inadequate or obsolete methodology, the impracticality of the knowledge provided, the lack of clinical practice that would help to reinforce knowledge in the long term, the lack of implementation of modern technology for dental shade selection in both universities, among others [41–43].

In the present study it was also observed that women were 55% more likely to have unfavorable attitudes towards tooth shade selection compared to men. This may have been due to the fact that women relied on their perception and accuracy for shade selection [14, 16]. In addition, a point to consider is that during the pandemic a significant number of women had multiple tasks in the family, work and/or academic environment, which probably made them concentrate on passing their courses in a global way and did not consider this clinical procedure as relevant, even more so when the clinical evaluations could not be performed directly on patients [16, 17, 44, 45].

The importance of the present study lies in the fact that dental students who are about to graduate should be familiar with the management of protocols for dental shade selection, even more so considering that esthetics is currently a priority for people who seek to achieve sociocultural integration in their environment [8]. Likewise, the identification of deficient knowledge and unfavorable attitudes will help dental teachers to be aware of the deficiencies in the cognitive, attitudinal and practical aspects in order to redirect learning strategies and thus contribute to their students developing adequate competencies in dental shade selection. In addition, the questionnaires of the present study could be used to identify problems regarding shade selection in dental students and professionals, since this topic can be complex even in experienced dentists, especially if they do not use modern tools for this procedure and taking into account that patients' expectations regarding esthetics are constantly increasing.

Among the limitations of the present study, we can mention that it was not possible to evaluate the respondents in person, since there were still restrictions due to COVID-19 at the time of the study [46]. Another factor that was not considered in the present study was the lack of evaluation of practices on dental shade selection. This was due to the fact that during the pandemic, teaching was performed remotely with restrictions for clinical procedures. Finally, the cross-sectional design used did not allow us to evaluate the dynamism of the knowledge of dental interns and its sustainability over time. It is advisable to conduct studies on the need and impact of dental shade science in the education of future dentists with longitudinal designs from the preclinical stage to the internship stage. In addition, it is also advisable to constantly include the use of updated technologies and techniques to obtain optimal esthetic results in patients.

## Conclusion

The majority of the dental interns presented insufficient knowledge and less than half had unfavorable attitudes about dental shade selection. In addition, being a student under 29 years of age was a risk factor for presenting insufficient knowledge, and at the same time was a protective factor for presenting unfavorable attitudes. Likewise, being a female student was a risk factor for presenting unfavorable attitudes about dental shade selection. It is important for students to acquire sufficient knowledge and develop favorable attitudes about shade selection, since it is fundamental for carrying out functional and esthetic dental treatments.

## Data Availability

All data analyzed during this study are available from the corresponding author on reasonable request (cesarcayorojas@gmail.com).

## Abbreviations

CI: Confidence interval
OR: Odds ratio
STROBE: Strengthening the Reporting of OBservational studies in Epidemiology
SPSS: Statistical Package for the Social Sciences
